# Biochar ageing improves soil properties, growth and yield of red radish (*Raphanus sativus*) in a Haplic Cambisol

**DOI:** 10.1371/journal.pone.0288709

**Published:** 2023-07-19

**Authors:** Patrick Nyambo, Hammond Motsi, Cornelius Chiduza, Mashapa Elvis Malobane

**Affiliations:** 1 Risk and Vulnerability Science Centre, University of Fort Hare, Alice, South Africa; 2 Department of Agronomy, University of Fort Hare, Alice, South Africa; 3 Department of Agriculture and Animal Health, University of South Africa, Roodepoort, South Africa; University of Minnesota, UNITED STATES

## Abstract

The use of biochar as a soil ameliorant has recently gained momentum. However, its application has been reported to have some adverse effects soon after the pyrolysis process. This study aimed to determine the effect of different biochar ageing methods and fertiliser applications on selected soil properties, growth, and yield of red radish (*Raphanus sativus* L.*)*. A 2 x 3 factorial arrangement was used in a complete randomised design (CRD) with three replications. The factors were (1) biochar ageing at three levels, i.e., naturally aged biochar (NB), artificially aged biochar (AB), and fresh biochar (FB), and (2) fertiliser at two levels viz fertilised (F) and non-fertilised (NF). A control treatment (without biochar) was also included. Irrespective of the ageing method used, biochar application significantly increased soil pH, while fertiliser application significantly reduced soil pH throughout the experiment. Similarly, biochar application significantly increased soil hydraulic conductivity compared to the control. However, after ten weeks, significantly higher soil hydraulic conductivity was reported in treatments with AB biochar compared to both NB and FB. The application of fertiliser in biochar-amended soils improves the soil’s hydraulic properties and increases radish growth. The study concludes that AB biochar + fertiliser application improves soil properties and growth of radish.

## Introduction

The unsustainable farming practices used by most smallholder farmers deplete the already low soil organic carbon (SOC) in South African soils [1,2]. As a result, the soils are highly degraded hence low agricultural productivity. To attain a sustainable agricultural system, researchers have, among other strategies, advocated for the use of biochar as a soil ameliorant [3]. The use of biochar as a soil ameliorant has recently gained momentum in South Africa [4]. Numerous studies have shown the potential benefits of biochar in improving soil’s physical [5,6], chemical [7,8], and biological properties [5,9]. However, its application soon after the pyrolysis process, herein referred to as fresh biochar, has been reported to have detrimental effects. For example, high hydrophobicity, which limits biochar interaction with nutrients, water, soil organic matter (SOM) [10], and localisation of nutrients, which becomes unavailable for plant uptake [11]. Therefore, there is a need for its optimisation to increase its effectiveness as a soil ameliorant. One strategy is to take the biochar through an accelerated ageing process.

Biochar ageing alters its physical, biological, and chemical properties, which in turn can significantly influence its interaction with the soil and organic matter [12,13]. During the ageing process, the decomposition of hydrophobic compounds introduces oxygen-containing functional groups with a high density of π electrons on the biochar surface [13,14]. These functional groups (mostly carboxylic, phenolic, and lactonic groups) are naturally acidic and result in a high surface charge density and cation exchange capacity in aged biochar [15]. As a result, nutrient retention, bioavailability, and mobility will be significantly improved [16], thus, increasing crop growth and yield [10]. Biochar can be aged naturally or artificially through oxidation, hydration, leaching, hydrolysis, freeze-thaw, wetting and drying cycles, mineralisation, and adsorption of dissolved organic matter onto biochar surfaces [16]. Natural ageing usually happens in the field over several years, decades or centuries, while artificial ageing is done in the laboratory using chemicals like hydrogen peroxide (H_2_O_2_), sulphuric acid (H_2_SO_4_), and HNO_3_ [16,17].

The different ageing techniques may result in inconsistent observations on the effectiveness of aged biochar as a soil ameliorant. For instance, Paetsch et al. [13] reported that water holding capacity effects were more pronounced in aged than fresh biochar, while Aller et al. [10] reported increased water content in fresh compared to artificially aged biochar. In a study by Hale et al. [18], fresh biochar increased soil pH while aged biochar reduced pH; similarly, Dempster et al. [19] reported an increase in pH on fresh compared to naturally aged biochar. On the contrary, Zhelezova et al. [14] reported that naturally aged biochar increased pH more than fresh. Fresh biochar increased the final maize biomass weight in the sandy loam and silt loam soils, whereas aged biochar only increased biomass weight in silt loam soils [10]. According to Thers et al. [20], there were no significant differences between naturally aged and artificially aged biochar application on oil grain, oil, and straw yield of seed rape biomass. In addition, the responses of biochar are specific to the site (soil and climate), feedstock, preparation method, conditions, and method of ageing; therefore, it is essential to evaluate the effects of biochar ageing under South African conditions for informed eco-specific recommendations.

Radish (*Raphanus sativus* L.) is a dicotyledon from the family of Brassica which is grown for human consumption and animal consumption and is used as a cover crop in multiple cropping systems and conservation agriculture [21,22]. In South Africa, it is mainly grown in the KwaZulu-Natal and Eastern Cape provinces [22]. Radish is a short-cycle, highly nutrient-demanding crop sensitive to poor hydraulic conditions [23]. Therefore, supplementing nutrients using fertilisers significantly improves root morphology, improving its capacity to hold soil and penetration to nutrient-deficit zones [24]. However, the rapid loss of fertiliser may compromise its growth and development, necessitating the need to include the co-application of fertilisers with organic amendments like biochar.

Besides biochar ageing, the application of blended biochar and inorganic fertiliser is becoming another dimension to manage the high hydrophobicity and localisation of nutrient challenges of biochar soon after application. The dynamics associated with such a combination is that inorganic fertiliser provides already available nutrients for immediate plant uptake. At the same time, biochar, through its high SOC and other properties, manipulates soil conditions to limit nutrient losses, improving nutrient use efficiency. However, there are still limited studies in this regard. One of the available studies indicated that biochar alone yielded 3.6–4 t ha^-1^, while biochar co-applied with fertiliser yielded 4.1–5.5 t ha^-1^ over a four-year maize trial [25]. Still, soil chemical properties (pH, and ammonium-N, available P and exchangeable K) did not differ between the co-application and biochar alone, except on NO_3_-N where the biochar and mineral fertiliser combination was higher than biochar alone [25]. Thus, there is still a need to explore how this co-application influences soil properties and crop yields, especially on a short cycle of crops such as radish. This study aimed to investigate the effect of differently aged biochar and fertiliser addition on amended soil’s chemical and hydraulic properties and the growth and yield of radish under a greenhouse.

## Methods and materials

### Soil preparation

The study’s soil was collected from the Fort Hare University Research Farm (32°47’43.3 "S 26°50’54.1"E) in Alice, Eastern Cape Province, South Africa. The IUSS Working Group WRB [26] classified the soil as a Haplic Cambisol. The mean annual temperature and average annual rainfall within the research farm are 18.1°C and 575 mm, respectively [2]. The field has been under continuous maize production for more than six years. The field is ploughed using a tractor before planting. The soil was transported to the University of Fort Hare glasshouse, where it was air-dried before being sieved through a 2 mm sieve size. The selected soil properties are shown in Table 1.

**Table 1:** Selected properties of the soil and biochar used in this study.

Properties	Soil
pH	6.2
EC (uS/m)	220
SOC (%)	0.69
Ash content (%)	-
Volatile matter (%)	-
Available phosphorus (%)	2
Exchangeable magnesium	87
Exchangeable calcium	193
Exchangeable sodium	20.29
Exchangeable potassium	71–139
Free iron	0.945
ESP	3.668

EC: Electrical conductivity; SOC: Soil organic carbon; -: Not determined.

### Experimental design and treatments

A 2 x 3 factorial arrangement was used in a complete randomised design (CRD) with three replications. The two factors were (1) fertiliser at two levels viz fertilised (F) and non-fertilised (NF), and (2) biochar ageing at three levels, i.e., naturally aged biochar (NB), artificially aged biochar (AB), and fresh biochar (FB). A treatment without biochar was included as a control, thus, giving 21 pots. The pots were spaced 1 m apart.

### Biochar preparation and biochar ageing

Pyrolysis. The biochar feedstock used was derived from dairy-cattle dung which was sourced from the University of Fort Hare Dairy Trust grazing paddocks. The fresh cattle manure was air-dried before pyrolysis. Pyrolysis was done using a muffle furnace following Githinji [27] procedure.Briefly, the dried cattle manure was pyrolysed for 30 minutes in a 5 litre container at 500°C in a muffle furnace and then allowed to cool down in the absence of oxygen in a desiccator. Thereafter biochar was sieved using a 2 mm sieve to obtain uniformity. A biochar sample was taken to the laboratory for characterisation (Table 1).

Ageing process. Biochar was aged naturally and artificially. Artificial ageing was done using the hydrogen peroxide (H_2_O_2_) method following Liu et al. [28]. Briefly, 54.17 g of biochar was mixed with one litre of H_2_O_2_ (5%) in 1.5 litre glass jars. The glass jars were put in an oven at 80°C with regular agitation (two to three times a day) until all water evaporated and dried. The biochar was then further dried at 105°C for 12 hours and allowed to cool. Natural ageing was done following Ren et al. [29]. Briefly, soil and biochar were mixed at a rate of 5% weight of biochar per weight of soil (w/w). Soil and biochar were thoroughly mixed by shaking in 5 kg plastic bags before being taken to the incubator. Deionised water was added to the biochar-soil mixture, and the moisture content was adjusted to 40% water-holding capacity. Constant moisture in all the pots was maintained by regularly taking measurements using an HH2 moisture meter (Delta-T Devices Ltd, Cambridge, England). All the pots were incubated at 28 C for 30 days while stirring with a glass bar every two days while ageing. After ageing, the mixture was freeze-dried for one day and then removed to reach room temperature.

### Planting and crop growth in the greenhouse

The pot experiment was conducted for a total of 70 days. Seven kilograms of plastic pots were filled with 5 kg soil-biochar mixtures, while control pots were filled with soil only. Before planting, all pots were watered and left to freely drain to achieve field capacity. Five seeds of radish (*Raphanus sativus*) were planted in each pot and later thinned to one seedling per pot soon after germination. A pressure-plate apparatus was used to determine the field capacity of the soil [5]; thereafter moisture measurements were done using the HH2 soil moisture meter (Delta T-UK) to determine the amount of water needed to keep the soil at field capacity. Basal fertiliser and top-dressing fertiliser were applied only in pots receiving the fertiliser treatment. Single super phosphate and muriate of potash were applied at planting as basal fertiliser at rates of 110 kg/ha and 65 kg/ha, respectively, following a recommendation by Imthiyas and Seran [30]. Lime-ammonium (LAN) (28% N) was applied as a topdressing fertiliser, five weeks after planting at 90 kg/ha.

### Data collection and measurement

Crop growth and yield parameters. After planting, the pots were checked every day to determine the number of days to emerge. Percentage germination was calculated based on hypocotyls that appeared above the soil’s surface and were calculated following Eq 1.

$$percentage\;germination=\frac{number\;of\;seedlings\;emerged/pot}{number\;of\;seeds\;planted/pot}X\;100\%$$ (1)

During the crop growth period, leaf area was determined following Eq 2 as suggested by Silva et al. [31]. The length and width of the youngest fully developed leaf was measured using a 30 cm ruler. Leaf width was taken at the widest part of the leaf.

LA=CxLxf (2)

Where LA = leaf area, in cm^2^; C = length, in cm; L = width, in cm; and f = correction factor for radish (0.57).

A digital vernal calliper was used to measure bulb diameter. After harvesting, the tape root and bulb length were also measured using a 30 cm ruler. Each plant was separated into shoots, bulbs, and roots, put in a labelled sampling bag, and weighed to determine fresh mass. Dry mass was determined after drying the soil samples at 65°C for 72 hours.

Soil chemical parameters. Soil samples were collected fortnightly for the determination of selected soil chemical properties. The samples were air-dried before being taken to the laboratory for analysis. Both pH and electrical conductivity (EC) were determined using a glass electrode pH meter (Crison Instruments, Spain) at a ratio of 1:2.5 (soil/water) [32].

#### Soil hydraulic parameters

The cumulative infiltration (I) and saturated hydraulic conductivity (k) were determined using a minidisk infiltrometer (Meter Group Inc., Washington, USA) following Zhang [33]. Subsequently, I and k were calculated following Eqs 3 and 4, respectively.

$$I=C_{1}+C_{2}\surd t$$ (3)

where C_1_ (m/s) and C_2_ (m/s^½^) are curve fitting parameters; C_1_ is related to hydraulic conductivity, and C_2_ is related to soil sorptivity. The hydraulic conductivity of the soil (k) is then computed by using the following equation:

$$k=\frac{C1}{A}$$ (4)

Where C_1_ is the slope of the curve of the cumulative infiltration versus the square root of time, and A is a value relating the van Genuchten parameters for a given soil type to the suction rate and radius of the infiltrometer disk.

### Statistical analysis

Analysis of variance (ANOVA) for a completely randomised design (CRD) was done using JMP version 15.0 statistical software (SAS Institute, Inc., Cary, NC, USA). The study was done over ten weeks; therefore, time was introduced as an extra factor during the analysis.

## Results

### Soil properties

pH and electrical conductivity. The two-way interaction of time x fertiliser was significant with respect to pH (P<0.001) and electrical conductivity (P<0.05), while two-way interactions of biochar ageing x fertiliser and biochar ageing x time were only significant (P<0.01) with respect to soil pH.

**Fig 1:**
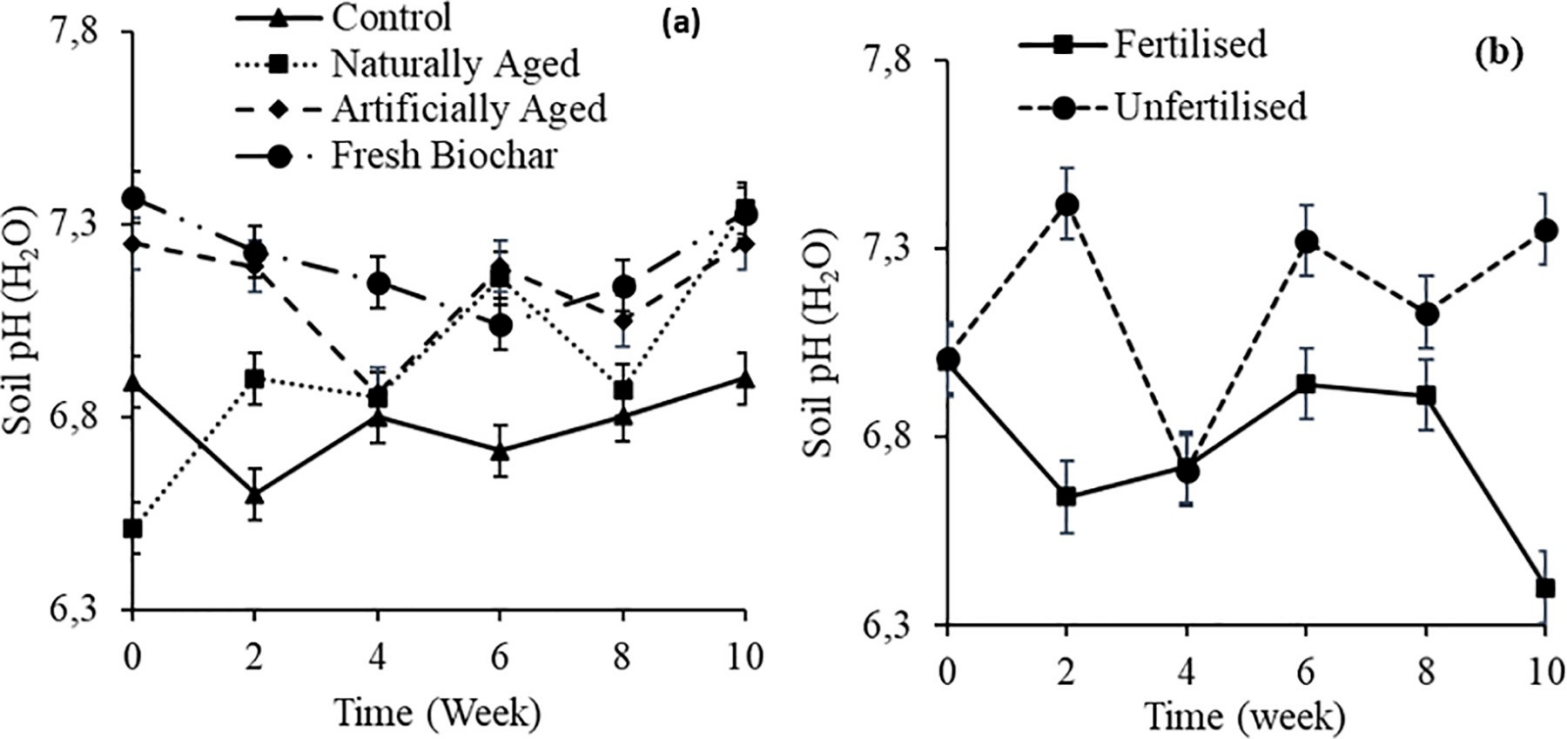
Interactive effect of (**a**) time x biochar ageing and (**b**) time x fertilisation on pH of a Haplic Cambisol soil. Error bars indicate standard deviation error.

On day 0, soil pH was significantly higher in soils amended with fresh biochar (7.37) compared to the control (6.89) and naturally aged biochar (6.51) (Fig 1**A**). From week 2 until week 10, the control treatment had a significantly lower soil pH than soils that were amended with biochar. Fertiliser application significantly reduced soil pH throughout the experiment except on week 4 (Fig 1**B**).

**Fig 2:**
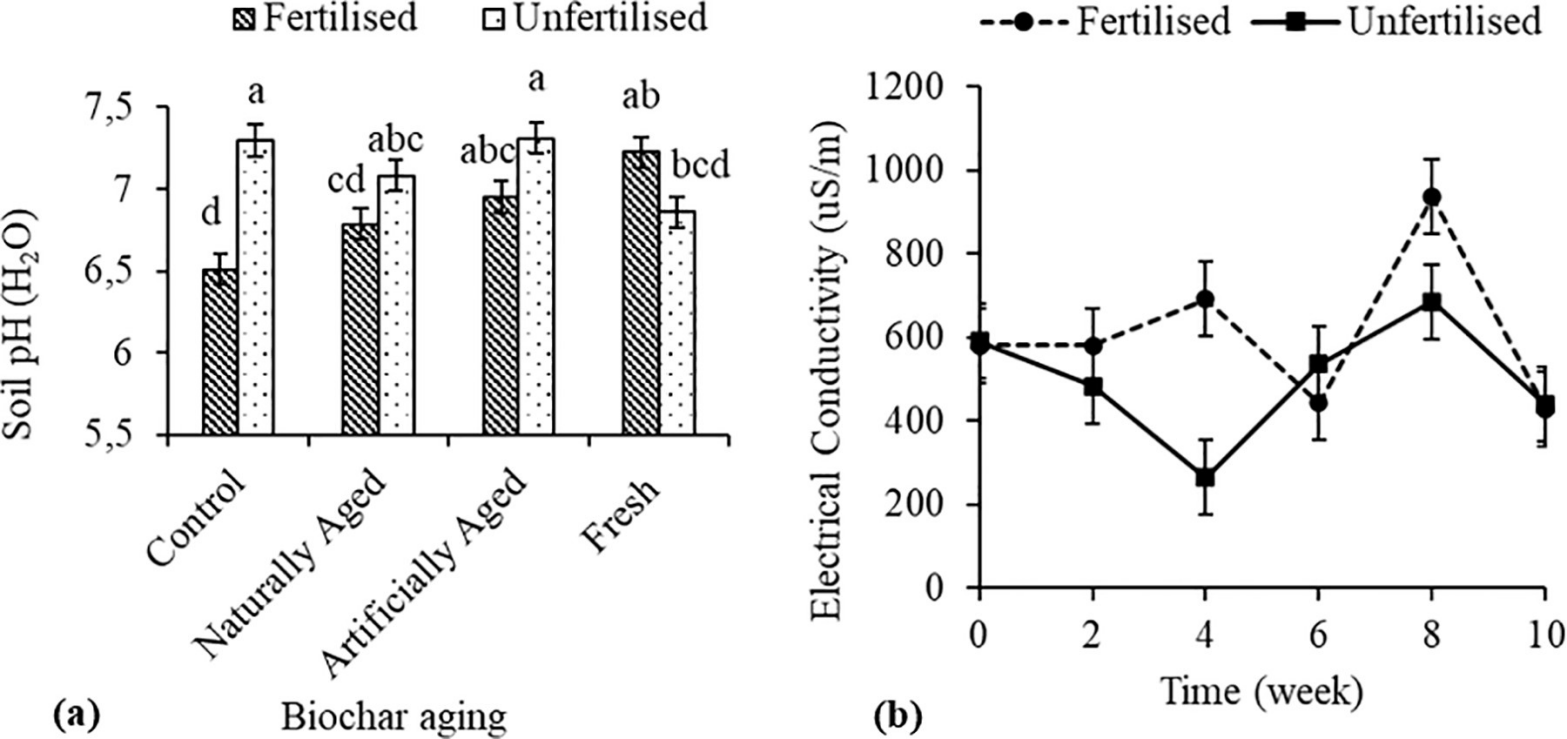
Interactive effects of (**a**) biochar ageing x fertilisation on soil pH and (**b**) time x fertilisation on the electrical conductivity of a Haplic Cambisol. Error bars indicate standard error. Different letters indicate significant differences among the treatments.

Fertiliser application significantly decreased soil pH (P< 0.001) by 11% compared to no fertilisers in the control treatment, while the interaction of fertiliser application and the various biochar-ageing treatments were not significant (Fig 2**A)**. Electrical conductivity was significantly higher in fertilised than unfertilised treatments in weeks 4 and 8 by 61.8% and 26.7%, respectively (Fig 2**B**).

Soil hydraulic properties. **Only the two-way interaction of biochar ageing x time was significant with respect to both infiltration rate (P<0.001) and saturated hydraulic conductivity (P>0.05).**

**Fig 3:**
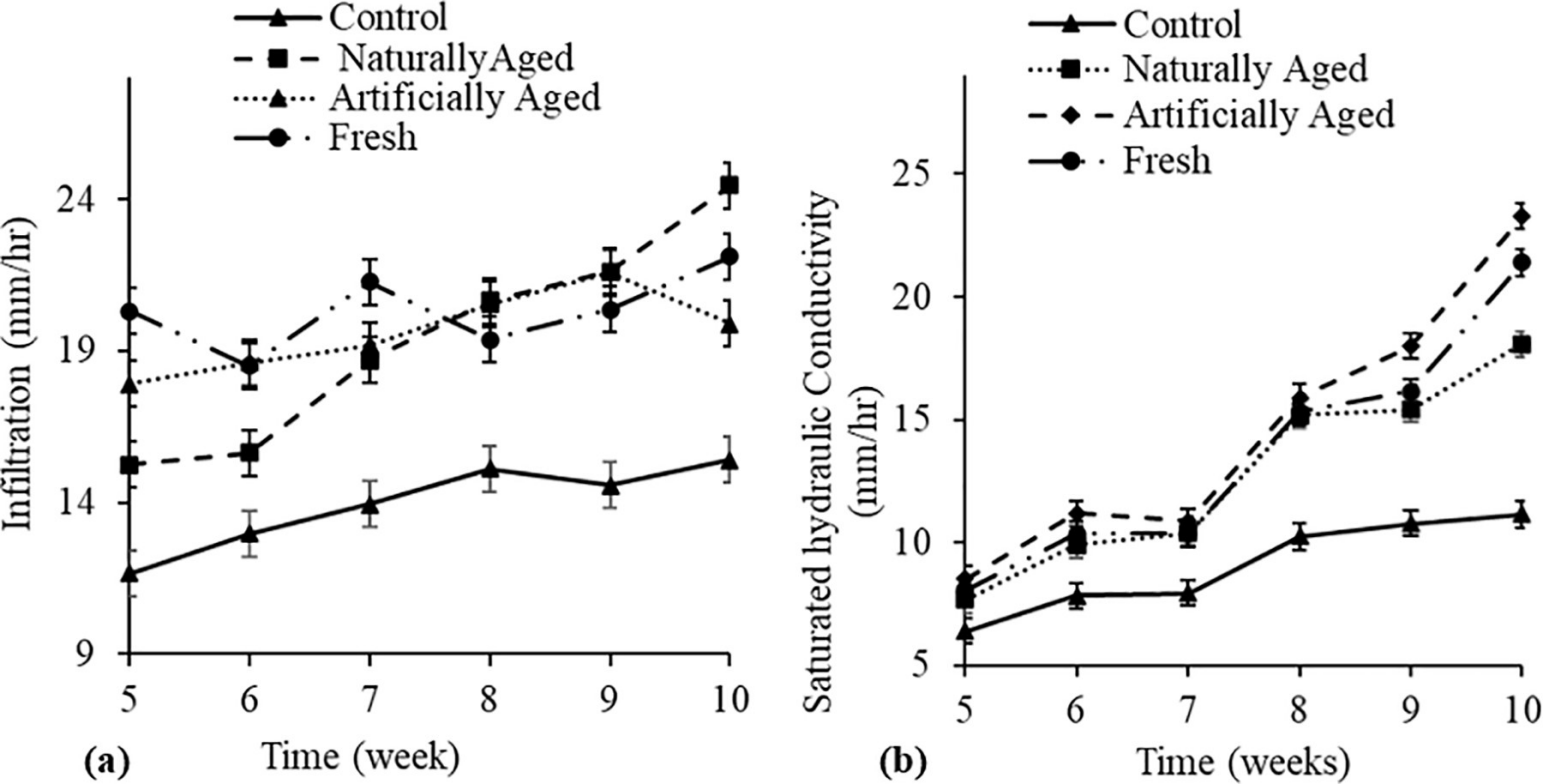
Interaction of time x biochar ageing on (**a**) infiltration rate and (**b**) saturated hydraulic conductivity of a Haplic Cambisol soil. Error bars indicate standard error.

Generally, the infiltration rate increased from week 5 to week 10 in all the treatments (Fig 3**A**). On week 5, infiltration was significantly higher by 42.4% in treatments with fresh biochar compared to the control treatment. At week 10, soils amended with naturally aged biochar had 37.1% and 18.8% higher infiltration rates compared to treatments with control and artificially aged biochar. Similar to the infiltration rate, hydraulic conductivity increased with time in all the treatments (Fig 3**B**). The control treatment had significantly lower saturated hydraulic conductivity compared to all the other treatments throughout the experiment.

### Plant growth

**Fig 4:**
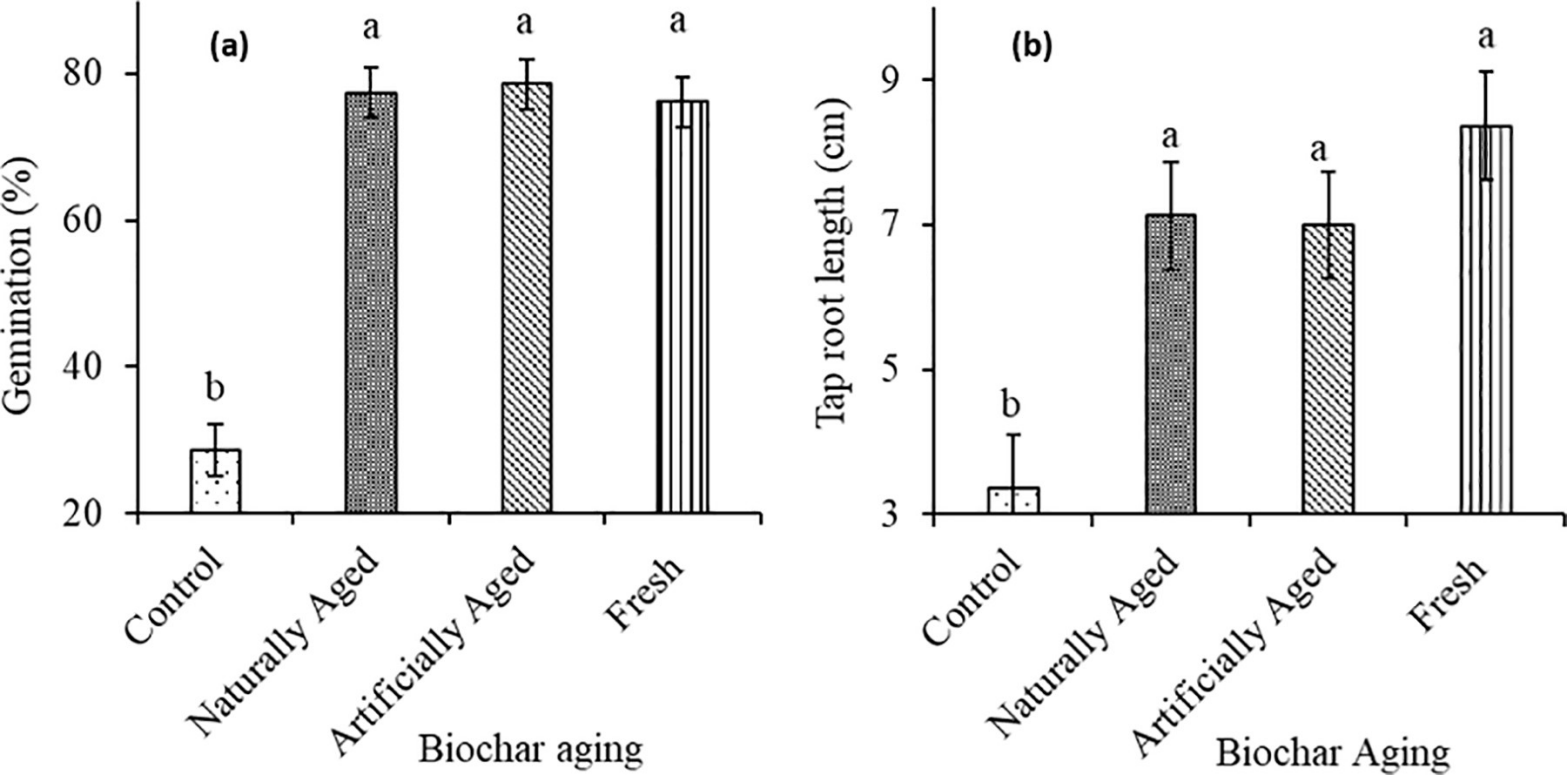
Effect of biochar ageing on (**a**) germination percentage and (**b**) taproot length of red radish in a Haplic Cambisol.

The various types of biochar ageing were not significantly different with respect to germination percentage and taproot length; however, they were all significantly (P>0.001) higher compared to the control (Fig 4**A** and 4**B**).

**Fig 5:**
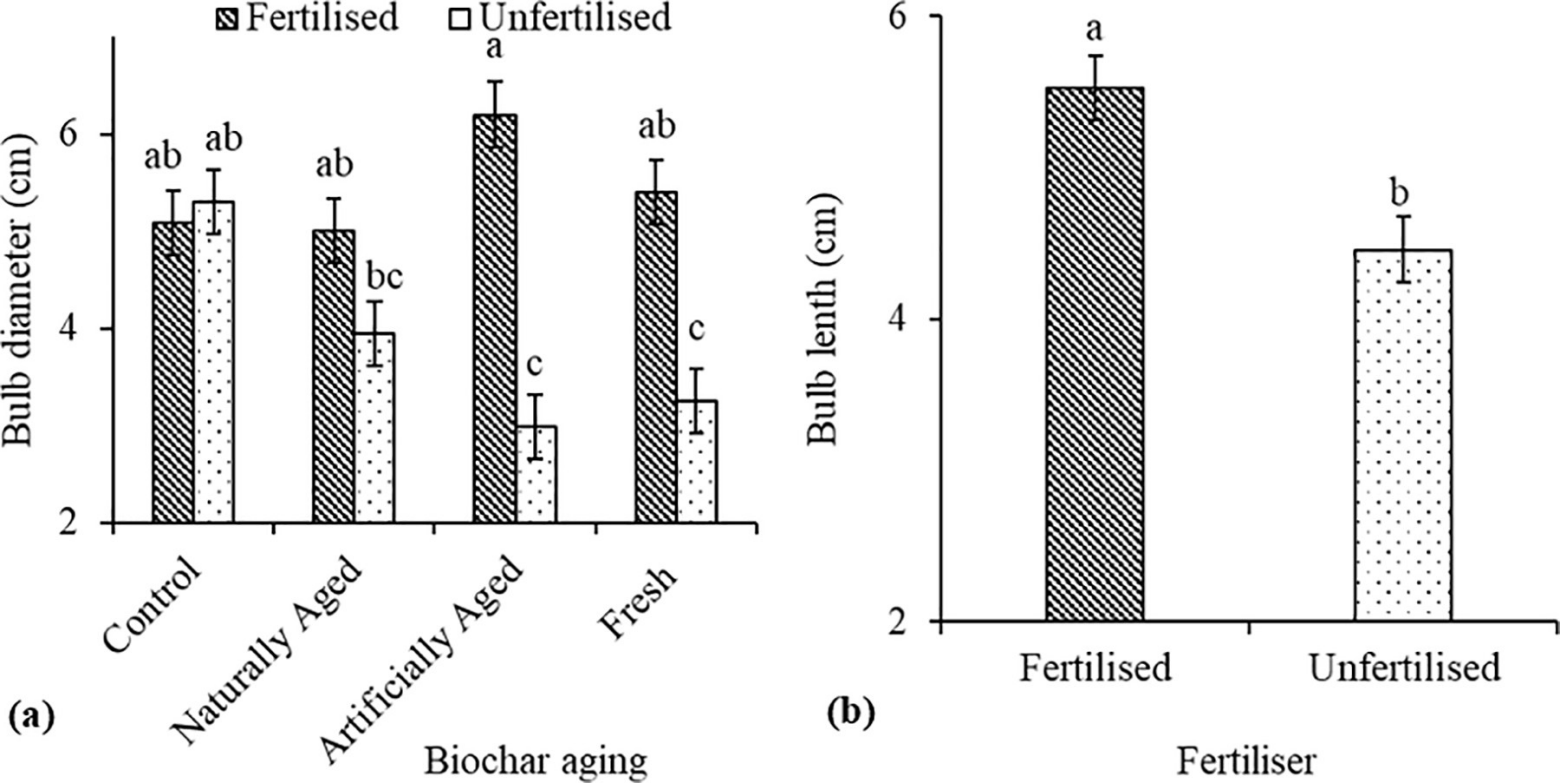
Interaction of (**a**) fertilisation x biochar ageing on bulb diameter and (**b**) effects of fertilisation on bulb length of red radish in a Haplic Cambisol.

Addition of fertiliser in treatments that had artificially aged biochar increased the bulb diameter of red radish by 51.6% compared to no fertilisation within the same biochar treatment (Fig 5**A**). The highest bulb length values were recorded in pots that were fertilised (53 cm) compared to the unfertilised pots (3.25 cm) (Fig 5**B**).

**Fig 6:**
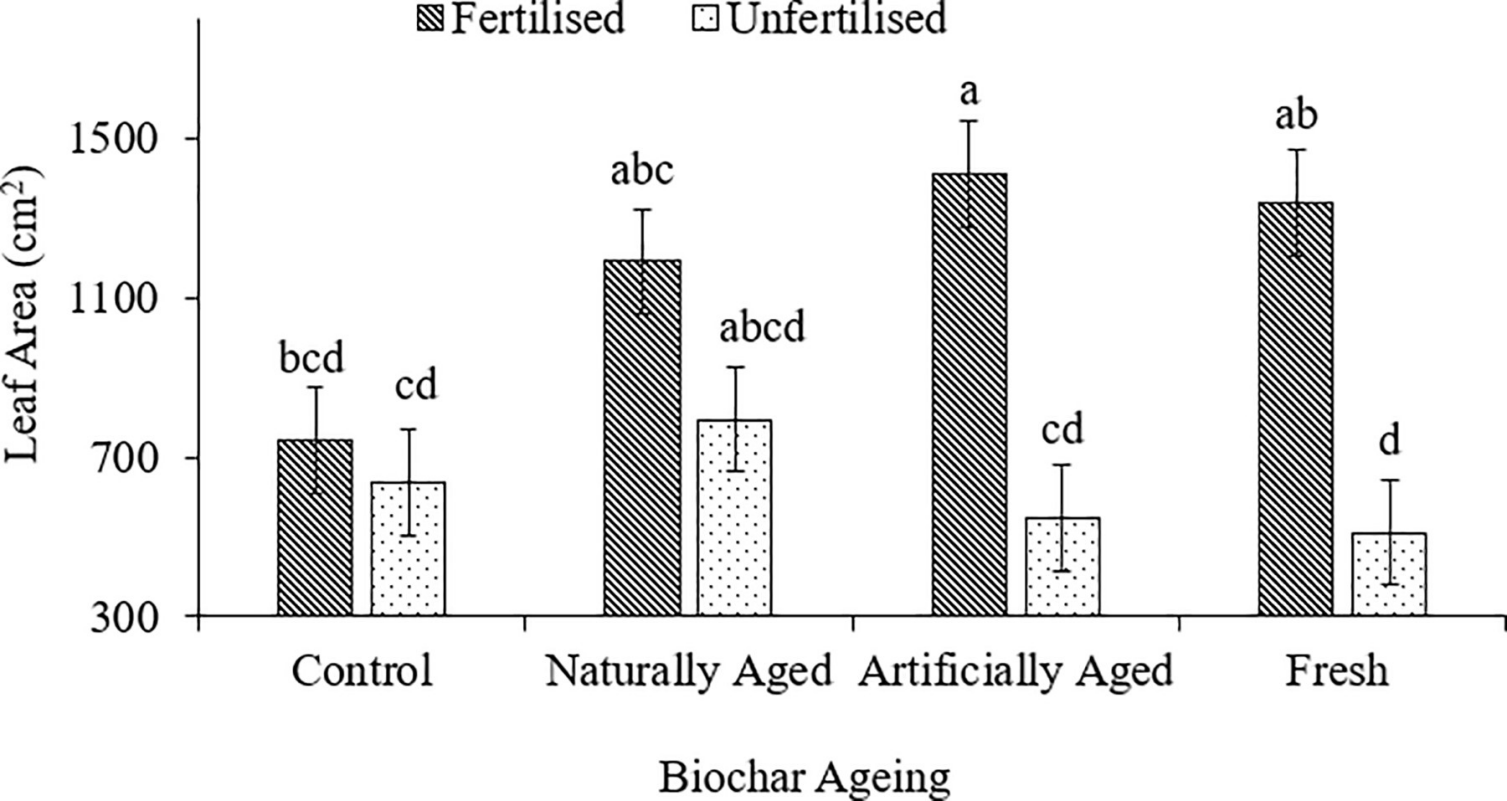
Effect of biochar ageing and fertilisation on leaf area of radish grown in Haplic Cambisol soil. Different letters indicate a significant difference.

Leaf area was significantly higher by 61.2% in fertilised pots compared to unfertilised pots under artificially aged biochar (Fig 6). Similarly, fertiliser application in artificially aged biochar treatment increased leaf area of red radish by 63.8% and 54.8% compared to unfertilised pots with the control and fresh biochar, respectively.

Fertiliser application significantly increased total biomass, root mass, bulb mass, and shoot mass irrespective of type biochar ageing (Table 2). The highest total biomass was recorded in treatments with fertiliser and artificially aged biochar, while the lowest was in treatments with the combination of artificially aged biochar and no fertiliser. Similarly, the treatment of artificially aged biochar and fertilisation significantly increased fresh and dry shoot biomass by 77% and 72.4%, respectively.

**Table 2:** Effect of biochar ageing and fertilisation on yield components of radish grown in a Haplic Cambisol soil. Different letters indicate significance difference.

		Total biomass		Root mass		Bulb mass		Shoot biomass	
		Fresh	Dry	Fresh	Dry	Fresh	Dry	Fresh	Dry
AB	F	196.66a	27.31a	2.73a	0.65a	117.85a	14.17a	73.51ab	12.44ab
NB	F	181.74ab	27.73a	2.51a	0.62a	80.22ab	10.66abc	98.7a	16.42a
FB	F	126.79abc	23.08a	1.55b	0.41bc	67.89ab	12.34ab	57.39bc	10.64bc
C	F	84.89bc	13.55b	1.66b	0.35b	37.86ab	5.2bcd	47.04bcd	7.93cd
NB	NF	79.41bc	11.56b	0.67c	0.2d	46.75ab	6.44bcd	29.11cd	4.93de
FB	NF	46.69c	7.46b	0.55c	0.16d	28.56b	3.88cd	19.62d	2.14e
C	NF	45.84c	7.07b	0.82c	0.12cd	23.08b	1.9d	27.54cd	4.49de
AB	NF	36.55c	6.76b	0.67c	0.12d	25.85b	3.45cd	16.73d	3.43e

AB; artificially aged biochar; NB: Naturally aged biochar; C: Control; FB: Fresh biochar.

## Discussion

The application of biochar enhances soil properties [34,35], and the ageing process affects the extent to which the soil properties are enhanced. Soil pH is a vital soil parameter that mediates nutrient availability, biological and chemical activities in the soil, and crop growth [36]. Applying all biochar types increased soil pH compared to the control without biochar. The finding is in line with many previous studies, which reported soil pH increases after applying biochar [5,37–39]. In this study, it is essential to note that both FB and NB amended soil had similar pH at the end of the study. This suggests that the applied natural ageing process in this study may have less influence on pH of biochar.

On the other hand, the difference in pH in soils amended with NB and AB confirms the reports by Ren et al. [29] and Tan et al. [40], who reported that biochar ageing processes affect biochar properties differently. The difference in pH between AB and NB treatment may also be because the artificial ageing process reduces biochar pH more than natural ageing [40]. Generally, the application of fresh biochar had higher pH throughout the study than aged biochar (Fig 1). This may be because, during the ageing process, carboxyl and hydroxyl functional groups increase on the biochar surface, which tends to reduce biochar pH [41,42].

The addition of fertiliser showed a decrease in pH and an increase in EC throughout the study compared to unfertilised (Fig 1). Fertiliser is known for increasing acidification and salts, which are some of its environmental shortcomings [43,44]. The application of biochar and fertiliser mixture had higher pH than the fertiliser application. This shows that biochar can buffer low soil pH [45]. The fact that applying biochar had no effect on EC does not correspond with previous studies that found biochar application to increase soil EC [37]. On the other hand, the results support the finding by Nyambo et al. [5], who also found no significant effect of biochar application on soil EC. This may suggest that its effect on EC is biochar specific.

This study found the lowest infiltration rate and hydraulic conductivity on the control throughout the study. This was also observed by Novak et al. [46], who reported an increase in infiltration rate after biochar application compared to treatments without biochar. The dynamics of how biochar improves infiltration rate and hydraulic conductivity arise from its properties such as hydrophilic nature, high internal porosity, high surface area, and relatively polar surface chemistry [17], which is due to the formation of oxygen-containing functional groups [47]. Thus, when biochar is incorporated into the soil, it positively influences soil properties, especially soil porosity which may allow smooth infiltration of water avoiding runoff and poor drainage. Qian and Chen [48] and Cao et al. [49] reported that biochar ageing increases the concentration of oxygen-containing functional groups on biochar surfaces that adsorb metal cations while making biochar surfaces more hydrophilic and hence less able to adsorb hydrophobic organic compounds. These properties are expected to improve [17]. For this reason, AB had a greater infiltration rate and hydraulic conductivity than fresh FB. This shows that AB was able to change the surface chemistry of biochar more than NB with ageing. According to Tan et al. [40], the artificial ageing process increases the specific surface area more than the natural ageing process. This may be why the infiltration rate and hydraulic conductivity were found to be higher under AB than in both NB and FB. Nevertheless, it is important to note that studies on how biochar ageing affects soil hydraulic properties are still scarce in the literature; thus, more research is still required.

Applying all the biochar types enhanced seed germination, which is in line with a study by Ke et al. [50] and Van Zwieten et al. [21], who also reported that radish germination increased with biochar application. The increase in seed germination after applying biochar may be due to the fact that biochar contains a variety of nutrients, such as nitrogen, potassium, sodium, calcium, and magnesium, that can influence seed germination [51,52]. In this study, aged biochar and fresh biochar did not show any significant difference, which shows that all the biochar created similar conditions required for radish germination.

Ke et al. [50] reported similar results as in this study by observing no difference in radish bulb length and width on biochar-amended soils compared to the control. On the contrary, Adekiya et al. [53] reported a significant increase in radish bulb length and width in biochar-amended soil compared to no biochar control. Thus, this warrants more research to fully understand the effect of biochar on radish bulb length and width. However, it is important to note that even though biochar had no statistical influence on radish bulb size in this study, the bulbs were generally bigger under biochar treatments than the control. Bulb length and width are affected by physicochemical properties (e.g., water holding capacity, bulk density, and porosity), fertilisation, and organic matter [52]. For instance, Adekiya et al. [24] reported a positive correlation between radish bulb length and porosity. Therefore, applying biochar could have improved the nutrient’s availability which may explain the bigger bulbs compared to the control treatment. This also possibly explains why fertilised pots had bigger bulbs than unfertilised pots.

Leaf area is important for the harvesting of light during photosynthesis. With a larger leaf area, more light can be captured and manufacture food for the plant. Silva et al. [31] supported this study’s findings and reported an increase in leaf area in radish after applying 2.8 g nitrogen per pot. Nitrogen is an essential nutrient for leaf development; therefore, adding nitrogen fertiliser provides the required nutrients for leaf growth. The application of biochar plus fertiliser treatments had a bigger leaf area than fertiliser only which is in line with the findings by Minhas et al. [53]. This can show that NB, AB, and FB treatments can supplement or enhance nutrient availability.

The high tap roots on fertilised compared to unfertilised treatments are in line with the study by Baloch et al. [52], who reported that radish taproot was high on fertilised compared to unfertilised treatments. This is because the addition of fertiliser supplied phosphorus which is responsible for the root growth. The difference between the control and all biochar plus fertiliser amended treatments could be due to the improvements in nutrient availability with the application of biochar. Aged and fresh biochar did not show any significant difference, meaning that the characteristics of aged and fresh biochar required for root development were similar.

The biomass yield increase after the application of biochar plus fertiliser is mainly attributed to the plants’ increase in nutrient availability and efficient use, especially nitrogen, which is of primary importance in biomass partitioning [54]. The increase in nutrient availability leads to an increase in more growth and development of plants, thus, increasing their biomass. The result in this study supports Baloch et al. [52] and Zafar-ul-Hye et al. [55], who reported an increase in dry and fresh weight after applying biochar plus fertiliser compared to the control (fertiliser only). Van Zwieten et al. [21] also reported a significant increase in biomass under biochar plus fertiliser blend compared to fertiliser only. AB plus fertiliser performed better than the other treatment combinations in this study. This shows that better yields can be attained by blending artificially aged biochar and fertiliser.

## Conclusions

This study aimed to investigate how biochar ageing and fertilisation affect selected soil chemical and hydraulic properties and the growth and yield of radish. The addition of fertiliser increased and decreased EC and pH, respectively. Fertiliser application did not affect the germination percentage of radish seeds, while biochar application significantly increased germination compared to the control. On the yield parameters, fertiliser-affected treatments have the highest numbers. Aged biochar (AB and NB) and fertiliser are effective for improving soil chemical and hydraulic properties and growth and yield of radish and can be recommended. However, further studies need to be done on how biochar ageing influences other soil properties and crops, which were not part of this study.

## Supporting information

S1 Data (DOCX)
