# Supplementary material for: Biochar ageing improves soil properties, growth and yield of red radish (Raphanus sativus) in a Haplic Cambisol
Source: PLoS One. 2023 Jul 19;18(7):e0288709. doi: 10.1371/journal.pone.0288709 (PMC10355431; doi:10.1371/journal.pone.0288709)
Supplement: S1 Data — (DOCX) [file pone.0288709.s001.docx]

**Table 1:** Summary of Anova for pH and EC.

| **Source of variation** |  | **pH** | **EC** |
| --- | --- | --- | --- |
|  |  | **(H_2_O)** | **(uS/m)** |
| Biochar Ageing (BA) | *p* | ns | ns |
|  | *f(3; 143)* | 2.2 | 2.5 |
| Time (T) | *p* | *** | ** |
|  | *f(5; 143)* | 6.5 | 5.3 |
| Fertilization (F) | *p* | *** | ns |
|  | *f(1; 143)* | 17 | 3.3 |
| BA x T | *p* | *** | ns |
|  | *f(15; 143)* | 3.1 | 1.4 |
| BA x F | *p* | ** | ns |
|  | *f(3; 143)* | 13 | 1.6 |
| T x F | *p* | *** | * |
|  | *f(5; 143)* | 5.9 | 2.8 |
| B x T x F | *p* | ns | ns |
|  | *f(15; 143)* | 1.7 | 0.8 |
| CV |  |  |  |

***, **, * and ns mean statistical significance at p < 0.001, < 0.01, 0.05 and nonsignificant, respectively.

**Table 2:** Summary of Anova for organic carbon (OC).

| **Source of variation** |  | **OC** |
| --- | --- | --- |
|  |  | **(%)** |
| B Ageing (BA) | *P* | ns |
|  | *f(3; 47)* | 1.1 |
| Time (T) | *P* | ns |
|  | *f(1; 47)* | 3.3 |
| Fertilization (F) | *P* | ns |
|  | *f(1; 47)* | 2.1 |
| BA x T | *P* | * |
|  | *f(3; 47)* | 6.1 |
| BA x F | *P* | ns |
|  | *f(3; 47)* | 0.2 |
| T x F | *P* | ns |
|  | *f(1; 47)* | 2.1 |
| B x T x F | *P* | ns |
|  | *f(3; 47)* | 0.2 |
| CV |  |  |

***, **, * and ns mean statistical significance at p < 0.001, < 0.01, 0.05 and nonsignificant, respectively.

**Table 3:** Summary Anova for Infiltration and Hydraulic Conductivity.

| **Source of variation** |  | **Infiltration** | **Hydraulic Conductivity** |
| --- | --- | --- | --- |
|  |  | **mm/hr** | **mm/hr** |
| B Ageing (BA) | *p* | *** | *** |
|  | *f(3; 149)* | 90 | 130.5 |
| Time (T) | *p* | *** | *** |
|  | *f(5; 149)* | 19.7 | 3.7 |
| Fertilization (F) | *p* | ns | ns |
|  | *f(1; 149)* | 0.7 | 1.6 |
| BA x T | *p* | *** | * |
|  | *f(15; 149)* | 4.0 | 3.5 |
| BA x F | *p* | ns | ns |
|  | *f(3; 149)* | 0.2 | 0.4 |
| T x F | *p* | ns | ns |
|  | *f(5; 149)* | 0.3 | 1.9 |
| B x T x F | *p* | ns | ns |
|  | *f(15; 149)* | 1.3 | 0.8 |
| CV |  |  |  |

*** and ns mean statistical significance at p < 0.001and non-significant, respectively.

**Table 4:** Summary of Anova for radish growth parameters.

| Source of variation |  | Gemination | Bulb diameter | Bulb length | Tap root length |  | Leaf area |
| --- | --- | --- | --- | --- | --- | --- | --- |
|  |  | % | cm | | |  | cm^2^ |
| Biochar Ageing (BA) | *P* | *** | ns | ns | * |  | ns |
|  | *f(3; 47)* | 50 | 2.6 | 0.8 | 9.3 |  | 0.1 |
| Fertilization (F) | *P* | ns | *** | * | *** |  | *** |
|  | *f(1; 47)* | 1.4 | 43 | 12 | 42 |  | 34 |
| BA x F | *P* | ns | ** | ns | ns |  | * |
|  | *f(4; 47)* | 3.2 | 9.7 | 0.4 | 2.6 |  | 3.8 |
| CV |  |  |  |  |  |  |  |

***, **, * and ns mean statistical significance at p < 0.001, < 0.01, 0.05 and nonsignificant, respectively.

Table 5: Data on the interactive effect of time x biochar ageing on pH of a Haplic Cambisol soil

|  | Control | Naturally Aged | Artificially Aged | Fresh Biochar |
| --- | --- | --- | --- | --- |
| 0 | 6,89abc | 6,51abc | 7,25a | 7,37a |
| 2 | 6,6bc | 6,9abc | 7,19a | 7,23a |
| 4 | 6,8abc | 6,85 | 6,86abc | 7,15ab |
| 6 | 6,713ab | 7,16ab | 7,19a | 7,04ab |
| 8 | 6,802abc | 6,87abc | 7,05ab | 7,14ab |
| 10 | 6,9a | 7,34a | 7,25a | 7,33a |

Different lowercase letters indicate significant differences at p < 0.001

Table 6: Data on the interactive effect of time x fertilisation ageing on pH of a Haplic Cambisol soil.

|  | 0 | 2 | 4 | 6 | 8 | 10 |
| --- | --- | --- | --- | --- | --- | --- |
| Fertilised | 7abc | 6,64d | 6,72bcd | 6,94abc | 6,91abcd | 6,4ab |
| Unfertilised | 7,01abc | 7,42a | 6,71bcd | 7,32a | 7,13abc | 7,35a |

Different lowercase letters indicate significant differences at p < 0.001

Table 7: Data on the interactive effects of biochar ageing x fertilisation on soil pH of a Haplic Cambisol.

|  | Fertilised | Unfertilised |
| --- | --- | --- |
| Control | 6,51d | 7,30a |
| Naturally Aged | 6,79cd | 7,08abc |
| Artificially Aged | 6,95abc | 7,31a |
| Fresh | 7,22ab | 6,86bcd |

Different lowercase letters indicate significant differences at p < 0.01

Table 8: Data on the interactive effects of time x fertilisation on the electrical conductivity of a Haplic Cambisol.

|  | 0 | 2 | 4 | 6 | 8 | 10 |
| --- | --- | --- | --- | --- | --- | --- |
| Fertilised | 580,13abc | 580,50abc | 691,50ab | 442,204bc | 936,24a | 427,81bc |
| Unfertilised | 590,96abc | 484,33bc | 263,94c | 535,92abc | 686,13abc | 438,79bc |

Different lowercase letters indicate significant differences at p < 0.05

Table 9: Data on the interactive effects of biochar ageing x fertilisation on the bulb diameter of a Haplic Cambisol.

|  | Fertilised | Unfertilised |
| --- | --- | --- |
| Control | 5,08ab | 5,3ab |
| Naturally Aged | 5ab | 3,95bc |
| Artificially Aged | 6,2a | 2,98c |
| Fresh | 5,4ab | 3,25c |

Different lowercase letters indicate significant differences at p < 0.01

Table 10: Data on the effects of biochar ageing x fertilisation on the red radish leaf area on a Haplic Cambisol

|  | Fertilised | Unfertilised |
| --- | --- | --- |
| Control | 742,18bcd | 637,99cd |
| Naturally Aged | 1190,73abc | 794,81acbd |
| Artificially Aged | 1410,83a | 547,2cd |
| Fresh | 1338,44ab | 511,39d |
